# How to Train Your Health: Sports as a Resource to Improve Cognitive Abilities in Cancer Patients

**DOI:** 10.3389/fpsyg.2019.02096

**Published:** 2019-09-13

**Authors:** Valeria Sebri, Lucrezia Savioni, Stefano Triberti, Ketti Mazzocco, Gabriella Pravettoni

**Affiliations:** ^1^Department of Oncology and Hemato-Oncology, Università degli Studi di Milano, Milan, Italy; ^2^Applied Research Division for Cognitive and Psychological Science, European Institute of Oncology IRCCS, Milan, Italy

**Keywords:** decision making, sport, cognitive functions, attention, oncology, psycho-oncology

## Abstract

From a cognitive-psychological perspective, physical exercise (PE) and sports are an interesting tool for improving people’s cognitive abilities. One field of application for such a tool is decision making (DM) support in chronic patients, cancer patients, and survivors in particular. On the one hand, cancer patients and survivors have to continually take important decisions about their own care (e.g., treatment choice; changes in lifestyle), in collaboration with caregivers and health providers; on the other hand, side effects of treatment may be detrimental to cognitive abilities, such as attention, which make the health DM tasks even more demanding, complex, and emotionally disruptive for patients. Since cancer patients have to engage in healthy activities both for improving their own quality of life and for sustaining the effects of medications, clinical advice to engage in sport and PE is becoming more and more widespread within interventions. However, while sports are usually seen as healthy physical activities, their impact on cognitive abilities is mostly overlooked in the literature. The hypothesis of the present work is that sports could be fully exploited in their potential as focused exercises for cognitive ability training, in the field of cognitive training for chronic patients specifically. Indeed, literature shows that different sports (e.g., individual or team-based) influence and possibly augment cognitive abilities such as focused and divided attention, working memory, and DM under time constraints. Moreover, besides providing training for cognitive abilities, the experience of sports may represent an opportunity to explore, train and sharpen DM abilities directly: we identify five ways in which sport experiences may influence DM processes, and provide indications for future research on the topic.

## Introduction

Oncological treatments influence patients’ physical and cognitive functions. Studies have provided evidence that cancer treatments, such as chemotherapy, radiotherapy, and hormonotherapy, can produce adverse effects including vascular complications, convulsion, mood disorders, and cognitive dysfunction (Alvarez et al., 2007; Dietrich et al., 2008). Many patients undergoing cancer treatment complain about so-called “chemobrain” (Brezden et al., 2000; McAllister et al., 2004), a cognitive decline associated with brain intoxication (Ahles and Saykin, 2001; Wefel et al., 2004; Butler and Haser, 2006).

Symptoms of “chemobrain” often persist after the completion of therapy and cause discomfort to survivors who are unable to return to daily life, finding difficulties at work and in other everyday life activities. Treatments have consequences not only in the post-treatment period, but also during the various phases of the disease, interfering with a patient’s ability to make good decisions. The cancer continuum is characterized by several stages: prevention, screening, diagnosis, treatment, survival or end of life. Each phase requires at least one specific decision (Reyna et al., 2015; Gorini et al., 2018). This process requires good cognitive skills and cognitive flexibility both to make the right choice *per se* and to make the appropriate changes in lifestyle in order to adapt to the impact of the disease and the side effects of treatment (Nelson et al., 2007; Arnaboldi et al., 2016; Triberti et al., 2019b).

Ideally, along this cancer continuum, decisions should be based on clear benefits and fully understood drawbacks, associated with an understanding of alternative courses of action (Reyna et al., 2015). The patient must be fully aware of what is happening to him or her, the current state of the disease, comparing all the different notions and ideas, and the multiple representations of illness and its consequences (Sainio et al., 2001; Renzi et al., 2016; Triberti et al., 2019a).

In light of the literature that demonstrated the importance of physical exercise (PE) and sport in preventing cognitive deterioration (Chu et al., 2015; Witte et al., 2016; Mandolesi et al., 2018; Walton et al., 2018), the objective of the present work is to investigate how this can be useful in cognitive training for chronic patients, cancer patients and survivors specifically. We will analyze those aspects of health-related decision making (DM) processes that can be enhanced by improving the cognitive functions involved in sport performance and experience. Then, we will suggest five ways in which sport experience could directly train DM, in order to inform sports-based health interventions for cancer patients.

## Decision-Making

Decision making, or how people make choices among available alternatives (Edwards, 1954; Thokala et al., 2016), plays a significant role in everyday activities. Studies have focused on understanding why people choose one option instead of another when choosing between a set of alternatives and identifying the processes behind it (Marasso et al., 2014). The amount of information people have access to increases over time, allowing them to reach more accurate decisions and stimulating critical thinking (Gréhaigne et al., 2001).

In order to understand DM, it is important to consider the continuous exchange between the Self and the environment. Perception is so divided between interoceptive and exteroceptive influences (Mirams et al., 2013). The awareness of optimal/unpleasant emotions or intrusive thoughts is an essential step for recognizing what are available resources and how to recruit them (Hanin, 2013), taking into account external distraction (e.g., noise) (Toner et al., 2015). Every agent is indeed influenced by cognitive and somatic reactions to both internal and external stimuli, which become the target of self-regulation processes (Birrer and Morgan, 2010).

According to these studies, superior ability in DM does not depend on an accurate knowledge of information and alternatives only, but also on a well-developed procedural knowledge base; rather than limiting to a mere selection of alternatives, DM relies on knowledge about the environment and oneself. Personal beliefs and values are involved decisions, especially those related to life-relevant choices (Gorini et al., 2016).

Decision making processes involve a number of cognitive abilities ranging from memory to perception; however, especially when considering life-relevant choices such as those related to health management, the employment of cognitive processes may be reduced. On the contrary, experiential characteristics and individual differences should be taken into consideration.

The next sections will consider sports as a tool to improve cognitive abilities relevant in DM, and then the possibility for sports to train DM activity directly.

## Sports and Cognitive Abilities Training

The impact that physical activity has on quality of life has always been known: the literature shows that regular physical activity favors the cardiovascular and musculoskeletal systems (Ploughman, 2008; Vincent et al., 2012; Thivel et al., 2018); helps to prevent certain diseases such as diabetes, obesity and cancer (U.S. Department of Health, and Human Services, 1996; Gao and Mandryk, 2012; Mandolesi et al., 2017, 2018); and promotes an improvement in mood and overall well-being (Coulson et al., 2008; Landi et al., 2010; Anderson and Shivakumar, 2013).

In addition to physical improvement, recent studies have shown the impact of sport and physical activity on cognitive functions. Studies on elderly subjects show that resistance exercises are a protective factor against cognitive decline, and in particular they favor preservation of executive planning and working memory (Gligoroska and Manchevska, 2012; Bherer et al., 2013; Kirk-Sanchez and McGough, 2013; Hsieh et al., 2016). It has been shown that cognitive functioning is empowered by physical activity in any age group (Anderson-Hanley et al., 2012; Benzing et al., 2016).

Research has shown specific improvements in executive functions (Tomporowski et al., 2008, 2011; Barenberg et al., 2011; Voelcker-Rehage and Niemann, 2013; Verburgh et al., 2014), mainly in children and adolescents (Verburgh et al., 2014; Donnelly et al., 2016). For example, Davis et al. (2011) tested the effect of about 3 months of regular aerobic exercise on executive functions of weight-bearing and sedentary students using fMRI, successful performance measures and cognitive assessment. The results showed that aerobic exercise improved cognitive performance and exercise dose-response benefits were found (Davis et al., 2011). Other acute benefits (i.e., short and temporary) were found in working memory; for example, Pontifex et al. (2009) evaluating 21 students showed that there is a shorter latency during a working memory activity which was performed immediately and 30 min after an acute period of aerobic exercise compared with the pretest (Riddle et al., 2005; Pontifex et al., 2009; Berridge and Devilbiss, 2011); other positive results regarding PE effects on cognitive abilities include concentration (Sibley et al., 2006; Gao and Mandryk, 2012) and duration of attention (Iuliano et al., 2015). Regarding neurobiological mechanisms, PE increases the level of neurotransmitters, which are theoretically responsible for neuroplasticity, neurogenesis, and neurotransmitters processes (Machado et al., 2015). In addition, endurance exercise leads to improved cardiorespiratory fitness (e.g., maximum oxygen uptake) that influences neutrophins, oxygen, and the stress-associated hormone cortisol. Improvement in neurotrophin level reduces the cortisol release and, as a consequence, the psychological stress response. These and other physiological changes are linked to prefrontal brain activation correlated with memory and cognitive control tasks. Moreover, neuroimaging research suggests that PE can indeed improve memory performance and cognitive control (Heinzel et al., 2018).

## What Cognitive Abilities Are Trained by Sports?

While a complete review of the relevant literature is out of scope for the present contribution, it is possible to outline the main cognitive areas affected by sports and PE. In general, any sport activity has both general and specific improvements in cognitive functions, based on their context. For example, knowing how to select the correct course of action is an important process in DM: being familiar with the duration of the training/competition, the goal and the physical sensations of fatigue and effort guide an athlete to an appropriate choice of play (Smits et al., 2014). So, athletes continuously decide “what to do” (action selection) and “how to do it” (action specification) to try making the perfect move in terms of technique and energy investment (del Campo et al., 2011; Smits et al., 2014) and to achieve a successful performance (Chamberlain and Coelho, 1993).

Cognitive flexibility is an important asset in sports: for example, long-jumpers do not execute a rigid-programmed pattern of stride lengths, instead they must assess each time what could be an optimal contact with the runway and the regulation of the length of the final stride for optimizing jump length (Craig, 2013). Therefore, the skill of assessing and regulating their own bodies in reference to the context each time is essential to obtain optimal results.

An improvement in working memory is found in athletes performing aerobic activities (Cassilhas et al., 2007; Diamond, 2015). This kind of PE, common to many sports, is associated with a faster cognitive processing speed (Hillman et al., 2005) and better performance in the ability of executive control (Ludyga et al., 2016). It is also associated with improved attention control (Shatil, 2013), executive control processes (e.g., inhibition and switching), linguistic verbal-auditory processing (Smith et al., 2013), and working memory (Maillot et al., 2012; Shatil, 2013). Research by Nagamatsu et al. (2013) found that both resistance training and aerobic training positively impacted on cognitive functioning and resulted in functional plasticity in healthy older adults, starting from the use of motor skills through tactical knowledge and DM (Práxedes et al., 2018).

Team ball games increase the ability to shift attention as a special perceptual skill, directing attention toward stimuli which initially appear as irrelevant. This kind of training also leads to the improvement of pattern recognition or the knowledge of situational probabilities (Abernethy and Russell, 2016). Many sensorial stimuli bombard athletes, who must consider the shared space and simultaneous participation of others, with a sort of uncertainty regarding the action of an opponent player (Práxedes et al., 2018). During the game, players must select and filter salient information by redirecting the focus of attention.

There are also instructions and rules that athletes must respect in the tactical DM of a team. For this reason, players do not pass the ball to obvious players (e.g., unmarked ones). Therefore, it is possible that players fail to find the optimal technical and tactical solution; in other words, concentration and attention are fundamental for players to be able to see the various opportunities during a specific moment of play (Memmert and Furley, 2016).

In collective sports, players have various roles, each with different requests and cognitive abilities in progress. For example, in soccer the goalkeeper tends to learn to wait longer with the scope of collecting more information about the ball’s direction, increasing attention orienting. This strategy helps him or her to guide actions, resulting in more saves, learning how and when to stop the ball. At the same time, players near the goalpost have to make the decision whether to try a shot at the goal or pass the ball to a nearby teammate, evaluating the situation and choosing the most functional action more or less immediately (Gréhaigne et al., 2001; Cotterill and Discombe, 2016). It is essential to know, for example, what makes a movement deceptive (Brault et al., 2010).

In volleyball, as showed by Montuori et al. (2019), team roles are associated with different required degrees of cognitive flexibility. The integration between visual perception and all the other information presented during the game converge to DM as an integrated process of elaboration during specific times.

Indeed it has been demonstrated that expert athletes have greater fixation on relevant tasks and more successful experiences in DM than beginners (De Oliveira Castro et al., 2016). Specifically, Craig (2013) affirms that a player’s decision is influenced by geometric and kinetic properties of the game that are, for example, a player’s eye height and how high he must jump. In this sense, it is not sufficient to assess the physical properties of the environment (e.g., time and height), but also the athletes’ perceptions of their own abilities. Visual-spatial attentional processing is, at the same time, increased and volleyball players have to train using perceptual-cognitive tasks constantly with high flexible attention (Alves et al., 2013).

In conclusion, not only context and circumstances make the difference; sports involve cognitive training as an essential part of performance. The domain of cognition especially involved in sports are: executive functioning, working and declarative memory (Morris, 2018), attention and processing speed (Gao and Mandryk, 2012; Iuliano et al., 2015; Prakash et al., 2015). Athletes are extremely committed to empowering these processes in order to improve their role within the competition and achieve optimal performance.

## Sports and the Relation Between Cognitive Abilities and Decision Making

There are several studies that show how good performances in sport are characterized not only by the efficient execution of tactical movements, but also by a high level of DM. Indeed, an athlete will never achieve a positive outcome of his tactical movement if the selected skill is inappropriate to the context and to the specific situation in which it is performed (Liu et al., 2013). Therefore, DM is an ability that could be improved and modified through deliberate practice and the development of skills (Abernethy and Russell, 2016). To demonstrate this, there are numerous studies that compare the DM abilities of more and less experienced athletes, showing how the first, placed in a specific sport/competitive context, tend to perform more efficiently than the others in various steps of the DM process (Baker et al., 2003); moreover, expert athletes make decisions more quickly and more accurately than novices (Bar-Eli and Raab, 2006; Faubert and Sidebottom, 2012; Hepler and Feltz, 2012; Renfree et al., 2014; Vaughan et al., 2019) and are reported to have more sophisticated mental representations and procedural knowledge (“action plans”) that help them to solve problems in a more intuitive and automatic fashion (Frank et al., 2013; Evans et al., 2017). In the end, elite athletes report a higher number of interoceptive stimuli during the action itself (Haase et al., 2015). In other words their self-awareness increases and so does performance management as a consequence (Toner et al., 2015) through the perception and continual monitoring of inner sensations.

Research comparing expert and novice athletes is useful to see how DM skills can be trained and possibly improved by continual sports practice; although it is not realistic to expect non-professional athletes to develop at a rate similar to elite ones, it is possible to prefigure the implementation of sport experiences to help people to train their ability to make decisions.

## What Benefits for Cancer Patients?

Studies show that cognitive performance can be improved by duration of moderate to vigorous physical activity, for example in breast cancer patients and survivors (Hartman et al., 2018). Peterson et al. (2018) increased memory performance and executive functions in cancer survivors through a 12-week aerobic exercise intervention. Evidences of benefits of high-intensity interval training for aerobic fitness and cardiovascular risk factors are emerging in cancer patients (Northey et al., 2019). Specifically, Zimmer et al. (2016) show improvements in executive functions, especially attention, cognitive flexibility and planning, after exercise.

A high degree of interdisciplinary cooperation must be implemented to integrate medical treatment and sports, but a rapid change in well-being is commonly observed in oncological patients (Baumann and Bloch, 2013) during different phases: (1) treatment, (2) adjuvant therapy, and (3) exercises supervised by accredited physiologists and/or physiotherapists. A number of studies support the idea of benefits of exercise for cancer survivors, underlining significant health improvements (Irwin, 2009; Schmitz et al., 2010; Spence et al., 2010). In this sense, sports could be adapted to individual characteristics of patients: it is not important to turn patients into high-level athletes, but to help them to benefit from PE health outcomes, and to avoid contraindications related to health status.

**FIGURE 1:**
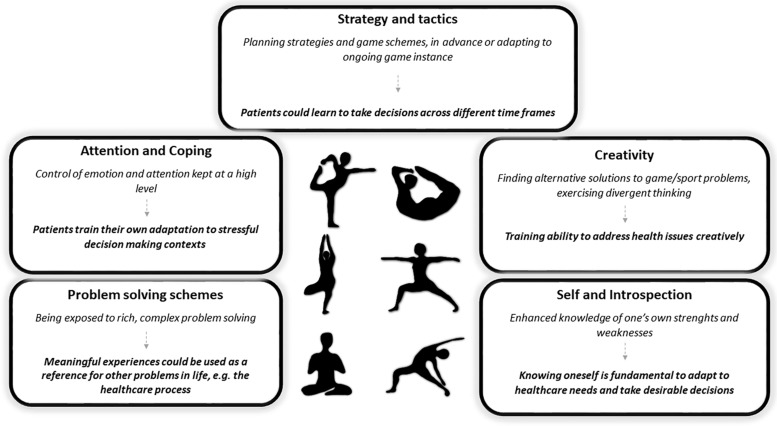
Five ways in which sport experience can lead to training of decision making abilities with a focus on cancer patients’ needs for health management.

We have seen how sports help to train cognitive abilities that are relevant to DM, possibly reducing the detrimental effects of the disease and side effects of treatment. We think that sports may share properties that help to train the ability to take decisions directly. At least five of such properties could be identified (resumed in Figure 1).

### Decision Making Training Through Strategy and Tactics

Strategical and tactical DM is a component of any sport, which many athletes would consider as important as physical preparation and technical skills (Renfree et al., 2014; Memmert and Roca, 2019).

Sports always include a pre-competition activity (formal or informal), in which athletes and coaches carefully plan the actions to be implemented later. For example, team sports require study of the opponents’ strategy and discussion of the choices and decisions in terms of team members placement and roles, and specific game actions to be enacted collectively or individually (e.g., deciding whether to conduct a more defensive or offensive match).

However, the actions previously planned must then be modified in the context of the competition, both by individual players and the team as a whole. This regards the necessity to implement tactical DM or being able to take novel decisions within a limited time frame, usually involving intuitive rather than rational thinking (Memmert and Furley, 2016; Memmert and Roca, 2019).

Cancer patients could benefit from the training of DM by sports activity. Sport provides the opportunity to train DM skills regardless of the sports context, thus allowing the patient to learn how to find possible solutions with others and then to implement them in his or her own situation.

Furthermore, training “tactical” and “strategic” DM allows one to both take decisions in the shortest time, an ability which should not be undermined when considering the importance of adhering to a therapy regimen (e.g., what should I do if I’m out of my medicine? Who should I call in case of emergency? etc.); and to forecast short and long-term consequences of choices, informing better DM processes in the present circumstance (e.g., will I be able to manage such a lifestyle change? Will my family do it too?)

It can be said that a patient’s situation is inherently different from that of athletes, and maybe that those are incommensurable: in particular, patients have time to make important choices while athletes take decisions in a short time, as the sport context demands. However, Herrmann et al. (2019) suggest that patients have not appropriate time to make care-relevant decisions; their perception of time may change often, in concurrence with situational factors (e.g., course of the disease) (Butow et al., 1997; Rodríguez-Prat et al., 2019), for example they feel “time is running out” or it is not enough to decide. Taking into consideration the subjective time, training the ability to decide in complex contexts could be a fundamental resource that patients may be able to implement through sports, along with exercising the ability to regulate emotions.

### Attention and Emotional Management

In any sport, athletes have to alternate focused and divided attention to monitor information important for managing the game or activity (Memmert, 2009; Liao and Masters, 2016). Moreover, such sophisticated attention management should often be maintained in the face of emotional activation, which could possibly be overwhelming and distracting (Corr, 2002; Jones, 2016). When in a disadvantageous position, or even when about to lose the game, a team and the individual players as well become able to control emotions and keep attentional functions at a high level.

Attention and emotional management, especially when trained together in a real-life context, are an important resource for taking desirable decisions and be confident in one’s own judgment. People who have to manage a chronic health condition could benefit from such an attention training which could be more effective than abstract cognitive exercises.

### Creative Decisions

Athletes very often have to find alternative solutions to the previously studied schemes, which do not fit to a specific situation; in such circumstances, creativity allows the athlete to “go beyond” basic rules and schemes in order to bring his or her own personal contribution to the athletic action, hopefully obtaining excellent results. This type of creativity is called “divergent thinking” or “tactical creativity” (Memmert and Roca, 2019); it refers to the ability to find the ideal, rare and flexible solution to a given problem.

For example, the great basketball player Earvin “Magic” Johnson is remembered for his so-called “*no-look-pass*,” which he used to deceive his opponents by looking in the direction of a free teammate while then passing the ball to another player (Memmert and Roth, 2007). The tennis champion Roger Federer invented the so-called “sabr” (which stands for “sneak attack by Roger”), a technique wherein he rushes in during the opponent’s second serve and takes the ball early.

These examples come from the world of high-level professional sport; however, they show how athletes approach problems with a creative stance, so that sport regulations, as well as PE *per se*, could be not limitations to problem solving but rather complex contexts that encourage divergent thinking and full expression of one’s own playing style (Colzato et al., 2013).

Thanks to tactical creativity athletes learn to re-elaborate usual practices and behaviors and to find solutions that no one had ever thought of before. In other words, sports allow the athlete to learn to go beyond what appears to be the initial information and rules, developing alternative solutions.

Finding alternative solutions that move away from predefined patterns is a very useful resource for patients who have to take decisions on therapy and lifestyle changes because it provides them with the ability to deal with the problem not being overwhelmed but finding solutions that may have positive implications.

### The Role of Self and Introspection

In sports, athletes may develop notable introspection processes. Indeed, they have to improve the knowledge about their own abilities, resistance to time pressure, personal characteristics, and strengths and weaknesses, in order to give their own contribution to the team and/or to develop their own personal playing style and approximate optimal performance. However, especially during the sport activity, they should be able to not be distracted by self-focused instead of performance-focused attention (Liao and Masters, 2016).

On the one hand, there is literature showing that introspection can negatively influence DM: “thinking too much” about one’s own motives and feelings could diminish systematic process of information and the capacity to discriminate between more and less important problem features (Wilson and Schooler, 1991; Tordesillas and Chaiken, 2007). However, especially for what regards life-relevant choices, self-knowledge is fundamental: people may attribute excessive salience to problem features, this way undermining their own peculiarity. For example, in a healthcare context, a patient may decide to change his dietary behavior. But then, the patient slowly discovers that he or she is not able to maintain the healthy diet in everyday life, so that the therapeutic process may be not effective in the end because of frequent violations of the rules the patient him- or herself had originally set.

By promoting reflections on one’s own capacity, as well as psychological introspection and metacognition, sports could help patients to learn how to take into consideration their own identity, personality, habits and peculiarities when facing important decisions, this way empowering their ability to manage their health status too.

### Transferability of Sport-Related Problem-Solving Schemes

Health professionals interested in using sports in interventions should appreciate that sports are not only tools to train physical and cognitive abilities, but also *experiences* that may have an important formative value, and profoundly influence athletes’ cognitive processes. When one has to win in a competition, he or she is driven to dedicate a notable amount of time to it, as well as cognitive resources even outside of the performances. For example, a boxer may mentally reproduce a fight in his or her own mind to forecast the opponent’s attacks and possible responses; a basket player may recall playing schemes as spatial mental representations to plan individual and team movements and actions. Indeed, imagery practices have been analyzed in sports both by experimental and anecdotal evidence (Behncke, 2004; Murphy, 2006; Schuster et al., 2011). Sport strategies and methods could root deeply as mental representations of problems and solutions, in accordance with situated cognition theories which sustain that our cognitive processes are based on real-life contexts and practices (Hutchins, 1995; Clancey, 1997). A patient who has to decide over therapy options or lifestyle changes and related struggles could represent decisions in a similar manner to the abstract representations coming from the sport experience, e.g., specific obstacles can be represented as an opponent team member to be dealt with at different times, with more or less risk, alone or with the help of other teammates, and so on; a climber could easily represent a healthcare journey as a climbing route, with different phases, more or less difficult, as time consuming and requiring some tools or others. Such mental representations are not poetic metaphors; rather, being based on subjective, meaningful experiences they could give indications on how to perform decisions in different fields, even that of one’s own health and disease management.

Such deep rooting of these mental representations tends to emerge only after long-lasting and dedicated experiences (Provvidenza and Johnston, 2009; Ennis, 2015; Bradley and Conway, 2016); this is another aspect of sports which should be taken into consideration and explored by health research, namely sports’ abilities in supporting problem solving representations and thus informing new personal approaches to health DM.

## Discussion

In conclusion, the practice of sport and more PE provides positive exercise for the body and mind as, in addition to preventing diseases such as heart disease and diabetes, they also increase cognitive functions and especially the executive functions, including the DM process. In oncological diseases, DM plays a key role as the patient is in the position of having to decide on important aspects that concern the entire continuum of the oncological disease, from prevention to end of life.

Improving cognitive functions through sport can be the first step to increasing psycho-physical well-being, especially during cancer treatment, as long as the possible medical limitations are taken into consideration; secondarily, we have shown how sports *experience* could constitute an occasion to explore, train and sharpen one’s own DM ability directly. Indeed, sport’s outcomes for well-being should not be reduced to simple byproducts of PE only.

Implications for future research mean accounting for the complex outcomes of sport experience implementations within chronic patients care, for cancer patients and survivors especially. It is possible to test the effectiveness of sport and PE in the empowerment of DM skills of cancer patients and survivors, investigating which type of sport is most suitable for this purpose, distinguishing between patients and their specific situations. Furthermore, future studies should explore the effects of sports on patients’ well-being after important healthcare decisions, in order to not reduce DM abilities to mere laboratory tasks, but instead analyzing its effects on everyday life and life-relevant choices. Systematic review efforts could be useful to identify evidence of transfer of sport-related experiences to DM in everyday life. In addition to quantitative research focused on outcomes, qualitative research could be employed to invite patients to narrate the experience of sports and the perceived transferability of sport skills and mental schemes to the management of healthcare decisions.

## Author Contributions

VS conceptualized the ideas presented in the study and wrote the first draft of the manuscript. LS contributed to the conceptualization and writing. ST supervised the writing and edited the manuscript. KM contributed important intellectual content and to the final revision. GP contributed important intellectual content and supervised the whole process.

## Conflict of Interest Statement

The authors declare that the research was conducted in the absence of any commercial or financial relationships that could be construed as a potential conflict of interest.
